# Housefly larvae hydrolysate: orthogonal optimization of hydrolysis, antioxidant activity, amino acid composition and functional properties

**DOI:** 10.1186/1756-0500-6-197

**Published:** 2013-05-17

**Authors:** Juan Wang, Yansheng Wang, Xiangli Dang, Xiaoxia Zheng, Wenqing Zhang

**Affiliations:** 1 State Key Laboratory of Biocontrol, School of Life Sciences, Sun Yat-sen University, Guangzhou 510275, China; 2 State Key Laboratory of Respiratory Disease, Guangzhou Institute of Respiratory Disease, The First Affiliated Hospital of Guangzhou Medical College, Guangzhou, 510230, China

**Keywords:** Housefly larvae, Protein hydrolysate, Antioxidant activity, Optimization, Two step enzymatic hydrolysis

## Abstract

**Background:**

Antioxidant, one of the most important food additives, is widely used in food industry. At present, antioxidant is mostly produced by chemical synthesis, which would accumulate to be pathogenic. Therefore, a great interest has been developed to identify and use natural antioxidants. It was showed that there are a lot of antioxidative peptides in protein hydrolysates, possessing strong capacity of inhibiting peroxidation of macro-biomolecular and scavenging free redicals *in vivo*. Enzymatic hydrolysis used for preparation of antioxidative peptides is a new hot-spot in the field of natural antioxidants. It reacts under mild conditions, with accurate site-specific degradation, good repeatability and few damages to biological activity of protein. Substrates for enzymatic hydrolysis are usually plants and aqua-animals. Insects are also gaining attention because of their rich protein and resource. Antioxidative peptides are potential to be exploited as new natural antioxidant and functional food. There is a huge potential market in medical and cosmetic field as well.

**Result:**

Protein hydrolysate with antioxidant activity was prepared from housefly larvae, by a two-step hydrolysis. Through orthogonal optimization of the hydrolysis conditions, the degree of hydrolysis was determined to be approximately 60%. Fractionated hydrolysate at 25 mg/mL, 2.5 mg/mL and 1 mg/mL exhibited approximately 50%, 60% and 50% of scavenging capacity on superoxide radicals, 1, 1-Diphenyl-2-picrylhydrazyl radicals and hydroxyl radicals, respectively. Hydrolysate did not exhibit substantial ion chelation. Using a linoneic peroxidation system, the inhibition activity of hydrolysate at 20 mg/mL was close to that of 20 μg/mL tertiary butylhydroquinone, suggesting a potential application of hydrolysate in the oil industry as an efficient antioxidant. The lyophilized hydrolysate presented almost 100% solubility at pH 3-pH 9, and maintained nearly 100% activity at pH 5-pH 8 at 0°C- 4°C and room temperature during the first 6 months of storage. Essential amino acids in the hydrolysate accounted for 43% of the total amino acids.

**Conclusions:**

The results suggesting that hydrolysate could be added to food oils as an efficient antioxidant. It might be useful for food additives, diet nutrients and pharmaceutical agents.

## Background

Antioxidants are one of the most important food additives that are widely used in the food industry. Currently, antioxidants are primarily produced by chemical synthesis, and they could accumulate in vivo and cause disease. Therefore, it is important to identify and use natural antioxidants. It has been shown that there are many antioxidative peptides present in protein hydrolysate that possess a strong capacity to inhibit both peroxidation of micro-biomolecules and free radicals [1-5]. Antioxidative peptides have the potential to be exploited as new natural antioxidants or functional foods. There is a great market potential in the medical and cosmetic fields for antioxidative peptides.

The functional properties of proteins can be improved by enzymatic hydrolysis under controlled conditions [6]. Consequently, enzymatic hydrolysis used for preparation of antioxidative peptides is a new hotspot in the field of natural antioxidants. Reactions occurred under mild conditions with accurate site specific degradation are repeatable and cause little damage to the biological activity of proteins. The substrates for enzymatic hydrolysis are usually plants and aquatic animals [4,7-17]. Some insects are also gaining attention because they are rich in proteins and bioactive peptides [18].

Housefly larvae, edible insects found in some areas of China, can now be easily reared on a large-scale with simple techniques and at low cost; however, they are commonly used for animal feed. In our laboratory, the antimicrobial peptides in housefly larvae have been isolated and identified [19,20]. The objectives of the present research are to obtain antioxidant peptides through enzymatic hydrolysis of housefly larvae, to study their amino acid compositions and functional properties, and to ensure a long lasting shelf life of the product.

## Results and discussion

### Optimization of enzymatic hydrolysis

Orthogonal experiment was designed to illustrate impact of different hydrolysis factors on DH of housefly larvae. To obtain hydrolysates with high DH and scavenging radical activity, the optimum hydrolysis condition was determined using the orthogonal experiment, the maximum DH of TSG could be obtained for hydrolysis conditions, it was confirmed the DH of two enzymatic processes were 18.01% (enzyme-to-substrate ratio, E/S) and 12.60% ( E/S) [13].

Bioactive peptides usually contain 2–20 amino acid residues per molecule [21]; the lower their molecular weights, the higher their chances of crossing the intestinal barrier and exerting a biological effect [22]. Previous work on antioxidative peptides had shown that peptides with 5–16 amino acid residues can exhibit potent antioxidant activity [3,23]. As a result, a two-step hydrolysis reaction was adopted, and orthogonal experiments were designed to optimize processing conditions to obtain smaller peptides, which might possess potent antioxidant activity.

The influence of pH (A), temperature (B), the solid/liquid ratio (C of first step), the amount of enzyme (D of first step, C of second step) and time (E of first step, D of second step) on the hydrolysis by Alcalase^®^ and Flavourzyme™ were determined using range (R) and ANOVA analyses. Statistical analysis results showed that among the independent variables, temperature, the solid/liquid ratio and the concentration of enzymes had relatively higher significant effects (p < 0.05) on hydrolysis as compared to pH, time and the interactions between factors.

**Figure 1:**
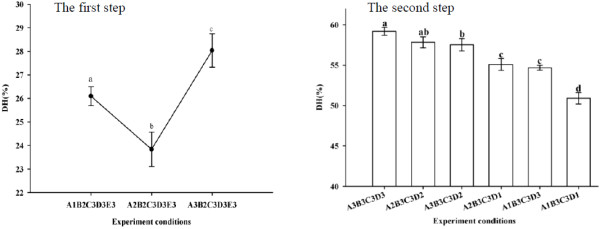
**Comparison of the degree of hydrolysis (DH) among several improved test conditions of the first step (the lines) and the second step (the bars) determined by orthogonal experiments. **Different small letters for each step indicate significant differences (Duncan’s new multiple-range test, p < 0.05). Five factors and three levels at each factor were listed in Table 2.

Because there was no significant difference between pH and time levels, another experiment was conducted to identify the best processing conditions for each step of hydrolysis, as shown in Figure 1. The hydrolysis conditions for obtaining the optimum DH in the first step were as follows: Alcalase^®^ 2.4 L at pH 8, 55°C, a solid to liquid ratio of 1/20, 2% protease, and a reaction time of 5 h. In the second step, using Flavourzyme™, the conditions were set to pH 7, 55°C, a solid to liquid ratio of 1/20, 200 U/g protease (based on the protein of substrate), and a reaction time of 8 h.

### Molecular weight distribution of hydrolysate

**Figure 2:**
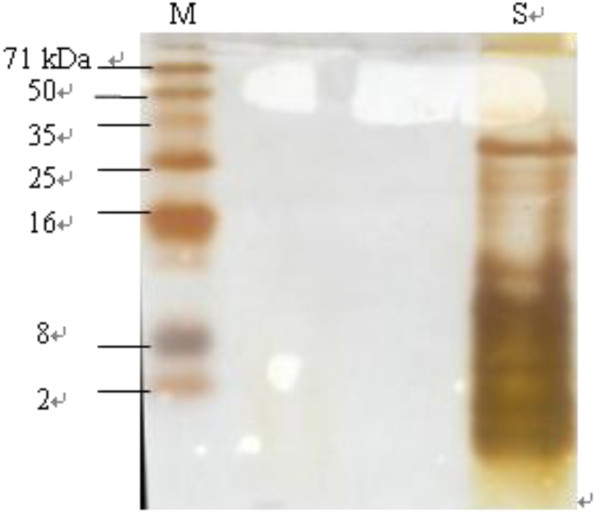
**Tricine-SDS-PAGE of hydrolysate purified by ultrafiltration. M**: protein molecular weight markers; **S**: sample of fractionated hydrolysate by ultrafiltration.

The electrophoretic pattern of the fractionated hydrolysate presented in Figure 2 indicated that the peptides formed due to hydrolysis were less than 16 kDa in molecular weights, corroborating well with the higher DH values observed in the study.

For the housefly larvae protein hydrolysate to be of high nutritional value, the dietary protein should be rich in low molecular weight peptides with the lowest possible amounts of free amino acids [24]. Hence, the protein hydrolysate prepared from the housefly larvae can be considered of high nutritional value.

### Antioxidant activity

#### Radical scavenging activity

Antioxidants activity depends on many different factors. We used three methods to characterize antioxidant activity based on the radical scavenging capacity: DPPH (1, 1-diphenyl-2- picrylhydrazyl) radical, the superoxide radical, and hydroxyl radical inhibition.

It is well known that the majority of free radicals are high biological activities while they exist only in short time, but DPPH can keep stable at room temperature. The DPPH free radicals are stable in ethanol and show maximum absorbance at 517 nm. DPPH radicals can interact with antioxidant, as a results, the radicals are scavenged and the absorbance is decreased. So, the DPPH radicals can be used to measure the scavenging activity of antioxidant compounds [25].

Superoxide radical, which is usually formed in cellular oxidation reactions, has a strong oxidation activity, it can produce singlet oxygen, hydroxyl radical, hydrogen peroxide and other strong oxidizing substances, it is very important in the initial reaction and the chain reaction of lipid oxidation [2]. Superoxide anion radical can cause damage to DNA and membrane of cell. So, it is very important to scavenge superoxide anion radical [2].

It is generally considered that the radical system could be used to evaluation antioxidant activity via radical-scavenging capacities of a antioxidant [26]. Hydroxyl radicals are the most reactive and can react with almost all the biomolecules in the cell, as a result induce damage to cells [27].

Statistical analysis results showed that among the independent variables, The superoxide radical, DPPH radical, and hydroxyl radical scavenging abilities of hydrolysate had relatively higher significant effects (p < 0.05) on hydrolysis as compared to Vc or TBHQ.

**Figure 3:**
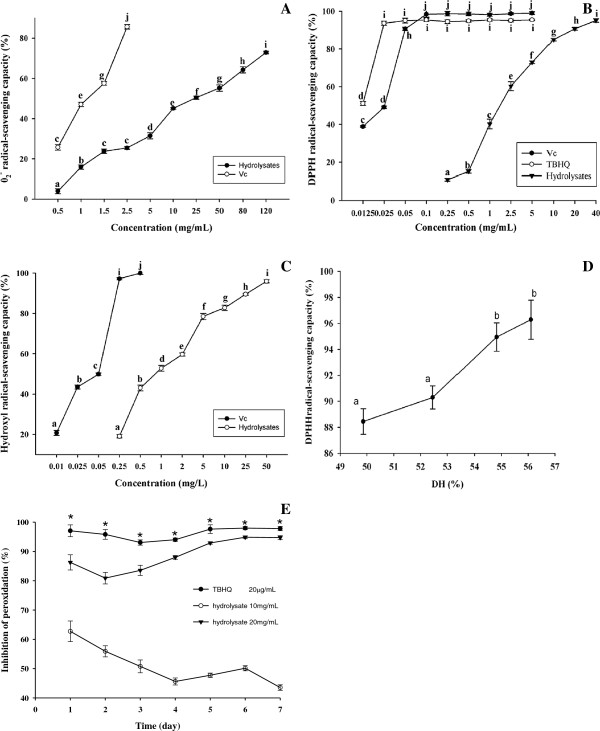
**Antioxidant activity of fractionated hydrolysate. A**. Superoxide radical scavenging activity of purified hydrolysates; **B**. DPPH radical scavenging activity of purified hydrolysates; **C**. Hydroxyl radical scavenging activity of purified hydrolysates. **D**. Effects of DH on the antioxidative activity of purified hydrolysates at 40 mg/mL. **E**. Effect of purified hydrolysates on the inhibition of linoleic acid peroxidation. Different letters within the same fold line indicate significant differences (Duncan’s new multiple-range test, p < 0.05).

The superoxide radical, DPPH radical, and hydroxyl radical inhibition of the fractionated hydrolysate of housefly larvae proteins were shown to be dose-dependent: the higher the hydrolysate concentration, the higher the superoxide radical, DPPH and hydroxyl radical scavenging capacity for the hydrolysate (Figure 3). The superoxide radical, DPPH radical, and hydroxyl radical scavenging abilities of hydrolysate at 10 mg/mL, 20 mg/mL and 50 mg/mL were similar to those of Vc at 1 mg/mL (Figure 3A), 0.05 mg/mL (Figure 3B) and 0.25 mg/mL (Figure 3C), respectively. The radical inhibition of hydrolysate was considerably lower than that of Vc or TBHQ at tested concentrations.

The IC_50_ value is applied as an indicator to evaluate the scavenging activity. The lower the IC_50_ value, the higher the free radical scavenging ability. The IC_50_ values for the scavenging ability of the hydrolysate for superoxide radicals, DPPH radicals and hydroxyl radicals were approximately 25 mg/mL, 2.5 mg/mL and 1 mg/mL, respectively (Figure 3).

In the present study, the hydrolysate did not exhibit substantial ion chelation.

**Figure 4:**
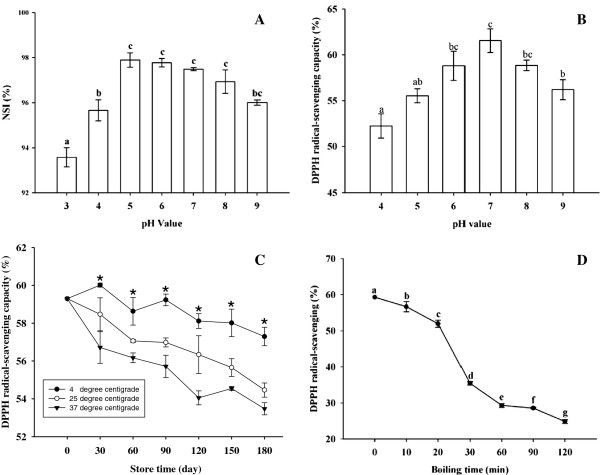
**Solubility and stability of the housefly larvae hydrolysate. A**. Effects of pH on the solubility of purified hydrolysates; **B**. Effects of pH on the antioxidative activity of purified hydrolyates. **C**. Effects of temperature on the antioxidative activity of purified hydrolysates; **D**. Effects of boiling time on the antioxidative activity of purified hydrolysates. Different letters within the same fold line indicate significant differences (Duncan’s new multiple-range test, p < 0.05).

Among all tested DH values, the higher the DH, the higher the DPPH radical scavenging capacity for the hydrolysate at 40 mg/mL (Figure 3D), indicating that, high DH may produce more antioxidant peptides. This DH-dependence of DPPH radical scavenging ability was found to be different from buckwheat [7], but the same as porcine collagen hydrolysate [28]. Some previous studies indicated that high DPPH or other radical scavenging activities for the protein hydrolysate or peptides are usually associated with highly hydrophobic amino acids or hydrophobicity [1,29], our results are consistent with those data (Table 1, Figure 4). The higher the concentration and the higher level of the DPPH radical scavenging activity were found in our experiment, similar to those results reported previously [30,31]. An isolated 1 kDa peptide from a peptic hydrolysate of casein showed superoxide anion as well as DPPH radical scavenging activity [32]. The scavenging activity against superoxide radical for wheat germ protein hydrolysates (0–0.60 g/L) ranges from 0% to 75.40% [31]. The scavenging activity against superoxide radical of the protein hydrolysate was lower than wheat germ protein hydrolysates, indicating effective chain breaking antioxidant of the protein hydrolysate.

**Table 1:** Amino acid compositions of fractionated hydrolysate and comparison with the FAO/WHO reference of essential amino acids requirements (mg per g of protein)

**Amino acids**	**Content of amino acids**		**Reference protein **^**d**^			
	**Hydrolysate**	**Larvae**	**1 year old infant**	**2-5 years old child**	**10-12 years old child**	**Adult**
Isoleucine*	52	34	46	28	28	13
Leucine*	78	55	93	66	44	19
Lysine*	51	68	66	58	44	16
Cystine	3	5	42^b^	25^b^	22^b^	17^b^
Methionine*	32	32				
Threonine*	54	38	43	34	28	9
Tryptophan*^a^	-	-	17	11	9	5
Valine*	74	61	55	35	25	13
Phenylalanine*	80	67	-	-	22^c^	19^c^
Tyrosine	43	70	-	-		
Histidine	9	24				
Aspartic acid	117	89	-	-	-	-
Serine	49	34	-	-	-	-
Glutamic acid	160	133	-	-	-	-
Glycine	47	39	-	-	-	-
Alanine	77	50	-	-	-	-
Arginine	16	41	-	-	-	-
Proline	37	36	-	-	-	-
Total amino acids	978^a^	877^a^				
Essential amino acids / %	43^a^	41^a^				

* Essential amino acids; ^a ^Tryptophan was not determined and counted due to degradation; ^b ^Sum of Cystine and Methionine; ^c ^Sum of Phenylalanine and Tyrosine; ^d ^Essential amino acids of reference protein according to FAO/WHO cited by Tang et al [33].

Antioxidative activity of protein hydrolysates also depends on the proteases and hydrolysis conditions employed [34].

#### Inhibition of linoleic acid peroxidation

Since lipid peroxidation is thought to proceed via radical mediated abstracted of hydrogen atoms from methylene carbons in polyunsaturated fatty acids [26], The inhibition of linoleic acid peroxidation by fractionated hydrolysate was measured as shown in Figure 3E. In the present experiment, the autoxidation of linoleic acid was inhibited to varying extents by different concentrations of hydrolysate (10 mg/mL, 20 mg/mL). The inhibition activity of hydrolysate at 20 mg/mL was similar to that of TBHQ (0.02 mg/mL), which was distinctly better than at 10 mg/mL. The underlying mechanism for the significantly different effect on inhibition in regards to concentration is still not clear, but it may be partially related to radical chain reactions.

Free radicals with a majority of reactive oxygen species (ROS) are usually unstable and react readily with other groups or substances in the body, resulting in cell damage and human disease [35]. Therefore, quenching of free radicals and ROS is probably one of the most effective defenses of a living body against various diseases.

### Amino acid composition of hydrolysate

Amino acid composition of the lyophilized fractionated hydrolysate was analyzed in order to determine the possible effect of the amino acid profile on antioxidant biological activity. Its amino acid composition is presented in Table 1. The total amount of amino acid is 978 mg per 1000 mg of sample. Some amino acids show antioxidant activity, such as Met, His, Cys and Tyr, His shows strong radical scavenging biological activity because its imidazole ring was decomposed [30,36]. Therefore, the antioxidant activities of the lyophilized hydrolysates seemed to be caused by these amino acids in the peptides. Moreover, the antioxidant activity of the lyophilized hydrolysates depended upon the amino acid sequence of the peptides. The analysis of the amino acid composition of fractionated hydrolysate showed that the content of essential amino acids accounted for 43% of total amino acids, and the equilibrium level fulfilled the suggested pattern of requirements by Food and Agriculture Organization/World Health Organization for children and adults.

The nutritional value of any ingredient depends on a protein’s capacity to fulfill the needs of the organism with respect to its essential amino acids.

### Functional properties of protein hydrolysate

Lyophilized hydrolysates were almost 100% soluble over a wide range of pH values (3–9) (Figure 4). Hydrolysate is known to have excellent solubility at a high DH [37,38]. Hydrolysis potentially influenced the molecular structure, hydrophobicity and polar groups of the hydrolysate [39,40]. Higher DH meant smaller peptides, which were expected to have proportionally more polar residues and the ability to form hydrogen bonds with water, thereby improving the solubility [38]. The Housefly larvae protein hydrolysates had a DH of approximately 60% (Figure 1), and fractionated hydrolysates consisted of low molecular weight peptides (Figure 2) that were mostly in the range of 2 kDa-8 kDa, which may support the findings of high solubility.

The high solubility of the housefly larvae protein hydrolysate over a wide range of pH indicated a substantially useful characteristic for additive applications. The characteristics of hydrolysate have been found to directly affect functional properties and uses as food ingredients [40].

Lyophilized hydrolysate was almost entirely active at pH 5-pH 8 between 0°C-4°C and at room temperature during the first 6 months of storage. After boiling for 30 min, the activity was significantly decreased (Figure 4).

## Conclusions

The two-step hydrolysis of the housefly larvae using Alcalase^®^ followed by Flavourzyme™ resulted in a degree of hydrolysis of approximately 60% under optimized conditions. A mixture of small peptides (2 kDa-8 kDa) was obtained after decoloration using activated clay and fractionation with ultrafiltration consecutively using 50 kDa and 10 kDa devices. The hydrolysates at 25 mg/mL, 2.5 mg/mL and 1 mg/mL exhibited 50%, 60% and 50% of scavenging activity on superoxide radicals, DPPH radicals and hydroxyl radicals, respectively. The inhibition of linoleic peroxidation by purified hydrolysate at 20 mg/mL was close to 20 μg/mL TBHQ, suggesting that hydrolysate could be added to food oils as an efficient antioxidant. In addition, hydrolysate is very nutritious and can be easily stored. In conclusion, hydrolysates might be useful for food additives, diet nutrients and pharmaceutical agents.

## Methods

### Materials

Housefly larvae were reared in our laboratory at 28°C±1°C, RH 60%-80%. The proteases employed for the optimization studies were Alcalase^®^ 2.4 L (2.4 AU/g) and Flavourzyme™ (1000 LAPU/g). Both proteases were purchased from Novozymes A/S (Gladsaxe, Denmark). Linoleic acid, 1,1-Diphenyl-2-picrylhydrazyl (DPPH), ferrozine and Vitamin C (Vc) were purchased from Sigma-Aldrich (Sigma-Aldrich Inc., St. Louis, MO, USA). Tertiary butylhydroquin-one (TBHQ) was purchased from TaiBang Co., Ltd (Guangzhou, China). Other chemicals and reagents used were of analytical grade and commercially available.

### Optimization of enzymatic hydrolysis

Orthogonal experiments were designed to optimize hydrolysis conditions and to obtain the highest amount of small peptides. Five factors (pH, temperature, time, solid/liquid ratio, and amount of protease) were varied at three levels for each factor, as shown in Table 2. The larvae were first hydrolyzed with endopeptidase Alcalase^®^ 2.4 L, followed by a mixture of endo- and exopeptidase (Flavourzyme™) to enhance hydrolysis and improve flavor. The orthogonal table, L_27_(3^13^), was used in both steps of the experiment. Degree of hydrolysis (DH) was measured for each test run and used as the index for optimizing the hydrolysis conditions.

**Table 2:** Factors and levels for orthogonal experiment design

**Factor**	**First step**			**Second step**		
	**1**	**2**	**3**	**1**	**2**	**3**
pH (A)	6.0	7.0	8.0	5.0	6.0	7.0
Temperature (B)	50.0°C	55.0°C	60.0°C	45.0°C	50.0°C	55.0°C
Solid/liquid ratio (w/v, C ^a^)	1/5	1/10	1/20	-	-	-
(enzyme-to-substrate ratio, E/S, D ^a^, C ^b^) ^*^	0.012 AU/g	0.024 AU/g	0.048 AU/g	50 LAPU/g	100 LAPU/g	200 LAPU/g
Time (E ^a^, D ^b^)	1.0 h	2.5 h	5.0 h	4.0 h	6.0 h	8.0 h

^a ^Factors for the first step of hydrolysis; ^b ^Factors for the second step of hydrolysis.

^* ^The protease is Alcalase^®^ 2.4 L at the first step, and Flavourzyme™ at the second step.

### Production and isolation of protein hydrolysate

Third instars’ maggots were dried at 80°C after washed, then the bound water was removed at 105°C, 12 h-24 h, the dried housefly larvae (1 g) were ground into a coarse powder and suspended in distilled water at a given volume. The hydrolysis reaction was initiated by the addition of 2.4 L Alcalase^®^ under the given conditions and was stopped by heating at 100°C for 5 min to deactivate the enzymes. The pH of the mixture was maintained constant during the hydrolysis by addition of 1 mol/L NaOH. After centrifugation at 10,000 g for 20 min, the supernatant was collected.

DH is the percentage ratio between the number of peptide bonds cleaved and the total number of peptide bonds in the substrate studied. DH was calculated as follows:

$$\begin{array}{l} \mathrm{DH}\left(\%\right)=\left[\text{content}\;\mathrm{of}\;\alpha -\text{amino}\;\text{nitrogen}\;\left(g\right)/\right)\\ \left(\text{content}\;\mathrm{of}\;\text{total}\;\text{analyzable}\;\text{Kjeldahl}\;\text{nitrogen}\;\left(g\right)\right]\times 100\% \end{array}$$

The content of α-amino nitrogen was determined according to United States Pharmacopeia [41], and the content of total analyzable Kjeldahl nitrogen was determined by a Kjeltec Auto 1030 Analyzer (Tecator AB, Sollentuna, Sweden) [42].

The supernatant was decolored by activated clay (8%, room temperature, 2 h) and was then filtered using a 0.22 μM filter membrane (Millipore, Massachusetts, U.S.A). To obtain low molecular weight peptides, the decolored hydrolysate was ultrafiltered with 50 kDa, followed by 10 kDa ultrafiltration devices (Millipore, Massachusetts, U.S.A). The fraction of proteins with lower molecular weights was collected, lyophilized to dryness in a vacuum, and used for the following experiments. The lyophilized powder was stored at -40°C before use.

### Molecular weight distribution of hydrolysate

The housefly larvae hydrolysates were analyzed by Tricine-SDS-PAGE according to [43] with slight modifications; 16.5% separating gel, 10% spacer gel and 4% stacking gel. The lyophilized hydrolysate was dissolved (20 mg/mL) in loading buffer (12% SDS, 6% β-mercaptoethanol, 30% glycerol, 0.05% Coomassie blue G-250, and 150 mmol/L Tris–HCl at pH 7.0), heated at 100°C for 5 min, and centrifuged at 12,000 g for 2 min.

The electrophoresis was performed in a mini electrophoresis apparatus (Bio-Rad, California, USA) at room temperature using the following procedure. The voltage was kept constant at 30 V until the samples completely left the stacking gel, then the voltage was kept constant at 90 V-100 V until the tracking dye was close to the bottom of the gel. The loading volume of the samples and the standards was 5 μL-10 μL. The protein bands were stained using silver nitrate according to the staining method used by [44]. The approximate molecular weights of the hydrolysate were determined using appropriate prestained protein molecular weight marker 2 kDa-71 kDa (SBS Genetech Co., Ltd, Beijing, China).

### Determination of antioxidant activity

#### 1, 1-Diphenyl-2-picrylhydrazyl (DPPH) radical scavenging activity

A method published by [45] was modified and used to test for DPPH radical scavenging activity. Briefly, 0.5 mL of 0.05 mg/ml the sample solution was mixed with 0.5 mL of freshly prepared 50 μmol/L DPPH in an ethanol solution. The resulting solution was then incubated at room temperature for 50 min in the dark prior to spectrophotometric analysis at 517 nm using a UV-spectrophotometer (Bio-Rad, California, U.S.A). A lower absorbance value at 517 nm indicated a higher DPPH scavenging activity. The capability to scavenge the DPPH radicals was calculated according to the following equation:

$$\begin{array}{l} \text{DPPH}\text{radical}-\text{scavenging}\;\text{activity}\;\left(\%\right)\\ \;=\left[{1-(A}_{S}{-A}_{0})/\left(A_{\text{control}}\right]\right)\times 100\% \end{array}$$

Variables A_s_, A_0_, and A_control_ represent the absorbance of the test sample, the blank sample without DPPH solution, and the DPPH solution without the test sample, respectively.

#### Ferrous ion-chelating ability

The Fe^2+^ chelating ability of the hydrolysate was determined according to the method reported by [46], with modifications. A test sample (250 μL of various concentrations) in phosphate buffer solution (pH 7.4, 925 μL) was treated with FeCl_2_ (2 mmol/L, 25 μL) and ferrozine (5 mmol/L, 50 μL) for 10 min at room temperature in the dark. The absorbance of the resulting solution was measured at 562 nm. A lower absorbance of the reaction mixture indicated a higher Fe^2+^ chelating ability. The chelating ability of the ferrous ions was calculated using the following equation:

Variables A_s_, A_0_, and A_control_ represent the absorbance of the test sample, the blank sample in which ferrozine was replaced by distilled water, and a control in which the sample was replaced by distilled water, respectively.

#### Antioxidant activity in the linoleic acid emulsion system

The thiocyanate method [47] was adopted to determine the antioxidant activity of the housefly larvae hydrolysate in a linoleic acid emulsion system as described by [48]. Each sample was diluted in 0.5 mL of absolute ethanol and mixed with the linoleic acid emulsion (2.5 mL, 0.02 mol/L, pH 7.0) in phosphate buffer (2 mL, 0.2 mol/L, pH 7.0). The linoleic acid emulsion was prepared by mixing and homogenizing 0.2804 g of linoleic acid, 0.2804 g of Tween-40 as an emulsifier, and 50 mL of phosphate buffer. The reaction mixture was incubated at 40°C in the dark. Aliquots of 0.1 mL were taken at several intervals during incubation. The degree of oxidation was measured according to the thiocyanate method by sequentially adding ethanol (4.7 mL, 75%), ammonium thiocyanate (0.1 mL, 30%), sample solution (0.1 mL), and ferrous chloride (0.1 mL, 0.02 mol/L in 3.5% HCl). After the mixture had rested for 3 min, the peroxide value was determined by monitoring the absorbance at 500 nm. A control experiment was performed with linoleic acid excluding the samples. The degree of oxidation was measured every 24 h until the absorbance of the control was constant. The lipid peroxidation inhibition was calculated using the following equation:

#### Hydroxyl radical scavenging activity

The hydroxyl radical scavenging activity of hydrolysate was determined according to the salicylic acid method [49] with some modifications. A solution containing salicylic acid in ethanol (9 mmol/L, 0.5 mL), an FeSO_4_ solution (9 mmol/L, 0.5 mL) and a H_2_O_2_ solution (9.8 mmol/L, 0.5 mL) were sequentially added to 0.5 mL of an appropriately diluted sample. The mixture was measured at 510 nm after reacting for 30 min in the dark. The hydroxyl radical scavenging ratio was calculated using the following formula:

Variables A_s_, A_0_, and A_control_ represent the absorbance of the test sample, the blank sample in which H_2_O_2_ was replaced by distilled water, and the control in which the sample was replaced by distilled water, respectively.

#### Superoxide anion scavenging activity

The pyrogallol autoxidation method was adopted according to [50] to test superoxide anion scavenging activity of the hydrolysate.

### Amino acid composition of hydrolysate

The analysis of the amino acid composition was performed according to [51]. The samples (30 mg) were hydrolyzed with 10 mL of 6 mol/L HCl. The solutions were sealed in tubes under nitrogen and incubated in an oven at 110°C for 22 h. then the hydrolysate was filtrated with filter paper, 1 mL filtrate was dried under vacuum conditions, after adding 1 mL deionized water to dried power and drying again, repeat this step, and the resulting dried material was reconstituted with a lithium citrate buffer at pH 2.2. The amino acids were quantified using a Hitachi L-8800 amino acid analyzer (Hitachi, Tokyo, Japan). Norleucine (Sigma–Aldrich, Inc., St. Louis, Mo., USA) was used as the internal standard. Hitachi 855–350 chromatographic column (Tokyo, Japan) was used in this experiment, temperature of column is 57°C, the temperature of the reaction column is 134°C, 20 μL sample was loaded into chromatographic column. Amino acid composition was reported as g amino acid per 1,000 g protein. Determinations were performed in triplicate and data correspond to mean values. Standard deviations were in all cases lower than 6%.

### Functional properties of protein hydrolysate

#### Solubility

To determine protein solubility, 250 mg of protein hydrolysate was dispersed in 5 mL of deionized water, and the pH of the mixture was adjusted to 3, 4, 5, 6, 7, 8, and 9 with 1 mol/L HCl or 1 mol/L NaOH. The mixture was stirred at room temperature for 2 h and centrifuged at 2000 g for 10 min. The Kjeldahl nitrogen in the supernatant and total Kjeldahl nitrogen of hydrolysate was determined by a Kjeltec Auto 1030 Analyzer (Tecator AB, Sollentuna, Sweden). Protein solubility was calculated as follows:

#### Stability of antioxidant activity

The antioxidant activity stability was studied using DPPH, the radical-scavenging method given by [45] with various pH and temperatures. To determine the stability in different pH environments, the lyophilized hydrolysate was dispersed in distilled water (2.5 mg/L) and the pH was adjusted to 4, 5, 6, 7, 8, and 9 with 1 mol/L HCl or 1 mol/L NaOH before adding protein (2.5 mg).

To determine the stability at different storage temperatures, lyophilized hydrolysate was stored at 0°C, 4°C, 25°C, and 37°C, with 60%-75% moisture. The DPPH radical scavenging activity was measured every 30 days for 180 days.

The hydrolysate (2.5 mg/mL) was boiled at 100°C for 10 min, 20 min, 30 min, 60 min, 90 min or 120 min. After cooling to room temperature, the DPPH radical scavenging activity was measured.

### Statistical analysis

In this study, each experiment was conducted in triplicate, and the values were expressed as mean ± Se, except that both orthogonal experiments were conducted once. The SAS^®^ system version 9.1 (SAS Institute Inc., Cary, NC, USA) was used for data processing. Analysis of variance (ANOVA) was performed. Differences in mean values were determined using Duncan’s new multiple-range test. Values of p < 0.05 were considered to be statistically significant.

## Abbreviations

AU: Anson unit; RH: Relative humidity; AP: Ammonium persulfate; D: Day; Da: Dalton; ddH2O: Double distilled water; DH: Degree of hydolysis; DPPH: 1,1-Diphenyl-2-picrylhydrazyl; G: Gram; g(rcf): Gravity(relative centrifugal force); kDa: Kilodalton; hr: Hour; M: Molarity(mol/L); min: Minute; N: Normality(g/L); OD: Optical density; Rpm: Revolution per minute; S: Second; SDS: Sodium dodecyl sulfate; SDS-PAGE: SDS-polyacrylamide gel electrophoresis; TEMED: N,N,N’,N’-Tetramethylene-Diamine; Tricine: N-(Tri(hydroxymethyl)) glycine; Tris: Tri(hydroxymethyl)-aminomethanetions.

## Competing interests

The authors declare that they have no competing interests.

## Authors’ contributions

JW designed the experiments, analyzed the data, drafted the paper, carried out the Optimization, Production and isolation, Determination, Amino acid composition, electrophoresis and functional experiments. YW contributed to the isolation, functional, Amino acid composition experiments and manuscript editing. XD and XZ contributed to Production and isolation experiments. WZ initiated the project, designed the experiments, revised the paper. All authors read and approved the final manuscript.
